# Identification of Plasmodiophora brassicae effectors — A challenging goal

**DOI:** 10.1080/21505594.2018.1504560

**Published:** 2018-08-26

**Authors:** Edel Pérez-López, Matthew Waldner, Musharaf Hossain, Anthony J. Kusalik, Yangdou Wei, Peta C. Bonham-Smith, Christopher D. Todd

**Affiliations:** a Department of Biology, University of Saskatchewan, Saskatoon, Canada; b Department of Computer Science, University of Saskatchewan, Saskatoon, Canada

**Keywords:** Clubroot, effectors, P. brassicae, Brassica, bioinformatics, pipeline

## Abstract

Clubroot is an economically important disease affecting Brassica plants worldwide. Plasmodiophora brassicae is the protist pathogen associated with the disease, and its soil-borne obligate parasitic nature has impeded studies related to its biology and the mechanisms involved in its infection of the plant host. The identification of effector proteins is key to understanding how the pathogen manipulates the plant’s immune response and the genes involved in resistance. After more than 140 years studying clubroot and P. brassicae, very little is known about the effectors playing key roles in the infection process and subsequent disease progression. Here we analyze the information available for identified effectors and suggest several features of effector genes that can be used in the search for others. Based on the information presented in this review, we propose a comprehensive bioinformatics pipeline for effector identification and provide a list of the bioinformatics tools available for such.

## Introduction

**Figure 1.**
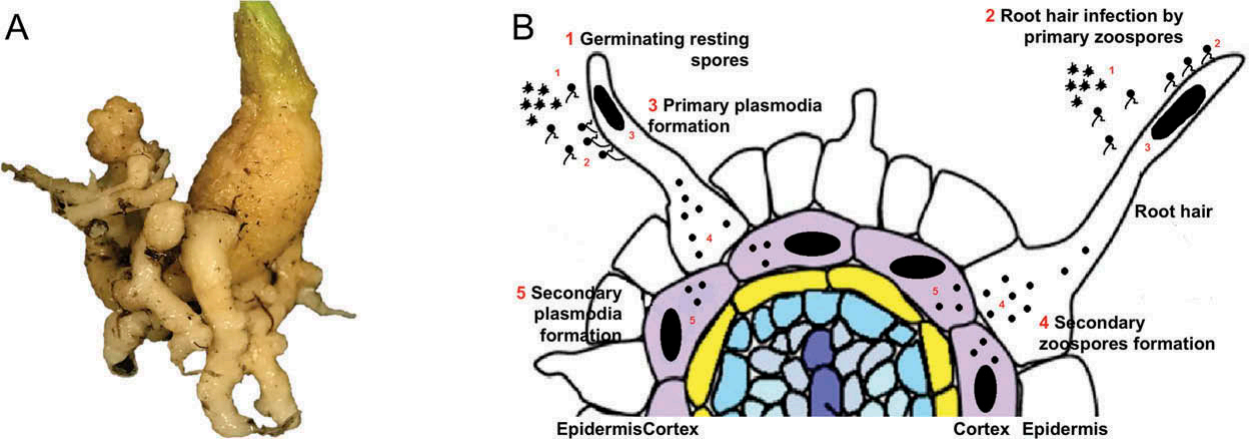
Brassica plant root affected by *Plasmodiophora brassicae*. A. Canola root with typical galls after 1 month of inoculation with *P. brassicae* resting spores. B. Life cycle of *P. brassicae* showing the steps involved in infection through to the production of secondary plasmodia in the host plant cortical cells. Scheme based on that of Kageyama and Asano [7], representing spindle-shaped resting spores, biflagellate primary zoospores, zoospores, and primary and secondary plasmodia (oval black figure in root hairs and cortical cells, respectively). Further steps in *P. brassicae*´s life cycle, such as the formation of resting spores in cortical cells and its ejection to the soil, are not shown in this scheme.

Clubroot disease is without doubt the most devastating disease affecting Brassicas, including the oilseed plant canola (*Brassica napus*) [1]. Brassica crops are widely cultivated and economically important for many countries around the world, with economic losses exceeding billions of dollars per year [1,2]. Clubroot disease, although it appears to have been first identified in Western Europe, today has been reported in countries as widely distributed as Brazil, South Africa, Australia, New Zealand, China, and Russia, across six of the seven continents [2,3]. Clubroot is caused by *Plasmodiophora brassicae*, a soil-borne pathogen member of the Order Plasmodiophorales, which are obligate intracellular parasites of fungi, algae, or higher plants [4]. In 2010, phylogenetic analysis also placed *P. brassicae* in the protist subgroup Rhizaria [5], one of the more poorly understood subgroups of the eukaryotes [5,6]. Plants affected by *P. brassicae* develop galls (abnormal outgrowths similar to tumors) on their roots to support the development of secondary plasmodia during the pathogen life cycle (Figure 1A)^7^. Gall formation leads to wilting associated with difficulties in water and nutrient uptake by the plant, and subsequent death [7]. Mature secondary plasmodia, the last stage of the pathogen life cycle, develop into resting spores that are released into the soil where they can resist severe environmental conditions for up to 20 years [7], making it almost impossible to prevent the disease through crop rotation and/or chemical treatments [1].

Breeding of clubroot-resistant cultivars is an important management strategy for controlling the disease, but in many countries such as Canada, there is a narrow genetic background with which to work [8–10]. Although there are some commercial clubroot-resistant (CR) canola and cabbage cultivars available, this resistance is associated with single dominant CR genes [11], as reported in Chinese cabbage [12] and oilseed rape [13], and leads to rapid breakdown. Two resistance genes have been isolated from Chinese cabbage (*Brassica rapa), CRa* and *Crr1* [14,15], while genetic mapping and identification of clubroot resistance has been also achieved in *Brassica oleracea* [16–19].

Most studies into the interaction between *P. brassicae* and its hosts have focussed on the plant, mainly because the pathogen is a soil-borne obligate biotroph impossible to study outside of the plant host. This pathogen life style is the reason why key knowledge pertaining to the identification of effector proteins mediating infection and subsequent disease progression is still unavailable. The recent draft genomes for European and North American *P. brassicae* pathotypes have provided the opportunity for the identification of putative effector proteins through comparative genomics [20,21]. However, low levels of similarity to known sequences at either the nucleotide and/or amino acid level has meant that annotating *P. brassicae* genes has proven to be extremely difficult.

Effectors from well-studied biotrophic plant pathogens such as the Basidiomycete rusts and the Ascomycete powdery mildews have been extensively studied and characterized as the basis of disease resistance breeding strategies [22]. Among these well-characterized biotrophic pathogens are: (*i*) *Puccinia monoica* [23] which, similar to aster yellows phytoplasma [24], can induce floral mimicry in order to promote its own sexual reproduction; (*ii*) *Melampsora lini* [25], which produces effector proteins in haustoria that are recognized inside the plant cell [26]; and (*iii*) *Cladosporium fulvum*, the tomato pathogen which, during infection, secretes the chitin-binding virulence factor Avr4 that is thought to protect the fungal chitin cell wall from hydrolysis by plant produced chitinases [27]. Extensive studies with *P. graminis* f. sp. *tritici* have identified race-specific avirulence factors (*Avr*) such as *AvrSr35* that mediates resistance against the highly virulent wheat stem rust race Ug99 [28] and AvrSr50, recognized by the Sr50 resistance protein, that provides resistance against all race groups of *P. graminis* f. sp. *tritici* worldwide, including Ug99 [29,30].

This review provides an overview of our current knowledge of putative effector proteins and suggests strategies for better annotation of the *P. brassicae* draft genomes with respect to effector proteins.

## What is known so far?

In order to manipulate plant defenses and enable parasitic colonization, many eukaryotic biotrophic plant pathogens have evolved advanced strategies to deliver effector proteins into the host cell during infection [31]. Successful infection relies primarily on the success of the release of the effectors, which in many cases are responsible for the suppression of plant immunity [32]. The initial recognition of conserved microbial features, known as pathogen – associated molecular patterns (PAMPs), leads to PAMP-triggered immunity (PTI) in the host [31]. PAMP-triggered immunity is a first level of immune response that can be overcome by effector proteins produced by adapted pathogens. Resistance (R) to adapted pathogens is achieved through specific recognition of effectors, also known as avirulence proteins, by corresponding R proteins produced by the plant host. This effector-R protein recognition constitutes the second level of immune response, effector-triggered immunity (ETI) [32]. *P. brassicae* is a well-adapted pathogen of *Brassica* hosts; though indirect evidence suggests lack of either response [33], both PTI and ETI have not been well-characterized in the host-*P. brassicae* pathosystem.

Understanding and identifying the proteins that comprise the secretome of *P. brassicae* is an important step towards identifying the complementary R proteins in the plant host. Putative effectors from biotrophic plant pathogens such as oomycetes and fungi are emerging from the sequencing and assembly of their genomes or transcriptomes, followed by comparative analysis with candidate effector genes [34,35]. This was precisely the strategy followed by Schwelm et al [20]. and Rolfe et al [21]. to identify putative effectors released by *P. brassicae* during the infection process. However, the obligate parasitic nature of *P. brassicae* has made it impossible to obtain a complete genome, with six draft genomes barely annotated for five Canadian pathotypes and one European pathotype [20,21]. Transcriptomes of *P. brassicae* infecting *B. napus* and *Arabidopsis thaliana* in the Canadian study, and infecting *B. rapa, B. napus*, and *B. oleracea* var. *capitata* in the European study, have identified some candidate effectors [20,21]. However, while the information obtained in both studies is a good start, the effectors specifically responsible for *P. brassicae* infection and subsequent disease progression have still to be identified.

A common finding in both studies was the over-expression of a benzoic acid/salicylic acid methyltransferase-encoding gene [PBRA_T000444 in *P. brassicae* European pathotype 3(Pbe3), PbPT3Sc00026_A_1.308_1 in *P. brassicae* pathotype 3 (Pb3) draft genomes; Genbank accession number AFK13134] during the second week of infection, with expression peaking three and four weeks after infection [20,21,36]. Salicylic acid (SA) is essential for the activation of plant defence [37]. In the plant cell, regulation of active SA is managed through the maintenance of different inactive forms of SA, such as methyl salicylate (MeSA) [38]. The methyltransferase identified in *P. brassicae* has been characterized in detail by Ludwig-Müller et al [36]., identifying its role in the methylation of salicylic, benzoic and anthranilic acids, thereby contributing to the suppression of the salicylic acid-induced defense in a plant host. This is the first and only well-characterized putative effector for *P. brassicae* and for this reason, we suggest that it is necessary to use a less conservative approach in this endeavour.

## Where and how to look for effectors?

The concept of an effector is constantly evolving with the understanding of plant-pathogen interactions. The basic criteria to identify candidate secreted effector proteins are: proteins with a signal peptide (within the initial 60 amino acids at the N-terminus), no trans-membrane domains, small size between 300 to 450 amino acids, and mostly species-specific [32,39,40]. These parameters were those used in the identification of putative *P. brassicae* effectors in the previously mentioned studies (Table 1). In addition, several other characteristics, motifs, and domains have been associated with effector proteins and have been used to improve identification and functional characterization of *P. brassicae* effectors.

**Table 1.** Steps to identify putative effectors within the secretome of *P. brassicae* in the European strain Pbe3 and the Canadian strain Pb3.

	Europe strain (Pbe3)	Canadian strain (Pb3)
Signal Peptide/No trans-membrane domain^a^	533*	590*
D score > 0.7	NA	431
Size (Small secreted proteins) ^b^	416	221
Over-expression ^c^	300	NA
Plant-free library ^d^	92	NA

NA, Not applied

^a^ In the Canadian strain, other subcellular localization signals were also used to remove putative proteins from further analysis.

^b^ For the European strain the cutoff was < 450 aa, while for the Canadian protein proteins < 300 aa were selected.

^c^ At least 10 expected fragments per kilobase of transcript per million fragments (FPKM)

^d^ FPKM log2 fold change > 5 in plant-free library

* Putative secreted proteins.

### Cysteine-rich proteins

Cysteine rich small proteins have been identified as effectors in several plant pathogens, especially fungi, such as *Cladosporium fulvum* (syn. *Passalora fulva*), an asexual extracellular fungal pathogen of tomato [41]. In *C. fulvum*, cys-rich effectors can inhibit and protect against plant hydrolytic enzymes, such as proteases, glucanases, and chitinases [32]. Cys-rich small-secreted proteins have also been identified as major effectors in the obligate biotrophic pathogens *Melampsora larici-populina* [35], and the Asian soybean rust fungus *Phakopsora pachyrhizi* [42], where one of the cys-rich small proteins identified as an effector has been shown to suppress plant immunity [43].

### Rxlr motifs

The motif RxLR, arginine-any amino acid-leucine-arginine, has been identified in the N-terminal of some oomycete and fungal effectors [43,44]. Although the function of the RxLR motif in effector proteins remains unclear, it has been shown to be necessary for translocation into the host cell [45] and to elicit immune responses in plant cells [46]. Curiously, despite the divergence between *P. brassicae* and oomycetes, RxLR motifs have been reported in effectors in both the *P. brassicae* Canadian strain Pb3 and the European strain Pbe3 [20,21], but the IDs of these RxLR protein-encoding genes have yet to be determined. Many putative effectors containing the RxLR motif also contain the second conserved motif, DEER (aspartate-glutamate-glutamate-arginine), located toward the C-terminus [47].

### Chitin-binding domains

Chitin, a recognized microbial PAMP, is a major structural component of fungal cell walls. Some fungal effectors have been shown to contain chitin-binding domains that are able to protect the pathogen against plant chitinases [48,49]. These effectors can also act as scavengers of chitin fragments released by the pathogen during infection [50], thereby avoiding a PAMP-triggered immunity response by the host plant. The resting spores of *P. brassicae*, formed at the end of the pathogen life cycle, contain chitin in their cell walls [20]. The presence of the carbohydrate/chitin-binding (CBM18) domain, enriched in the plasmodiophorid secretome, suggests that these putative effectors might be involved in the formation and possibly the germination of resting spores. A blastp (https://blast.ncbi.nlm.nih.gov) search with a chitin-binding domain protein, identified in the genome of Canadian strain Pb3 (PbPT3Sc00048_S_5.266_1), showed identity with a *Fusarium fujikuroi* chitinase (Genbank accession number CCT72994) [15]. In the Pbe3 genome, chitin recognition proteins, like the protein encoded by the PBRA_002543 gene, have also been identified [20].

### Protease/protease inhibitors

Sequence identity between plant pathogen effectors and other protein sequences is often low such that the assessment of functions based on putative orthology alone has been limited [31,50]. In many cases, the three-dimensional structure of the protein, the disulfide bond pattern, and the cysteine spacing have been used to identify protease and/or protease inhibitors as putative effectors in obligate biotrophic soil-born plant pathogens [34,51,52]. These effectors target host proteins/proteases during infection, thereby manipulating the host response to infection. During resting spore formation, Pbe3 overexpresses Kazal-like (e.g. PBRA_001430) and papain protease inhibitors [20]. The Kazal family of serine protease inhibitors, characterized by the presence of ten cysteine residues including the characteristic CX7CX6YX3CX2–3C signature, have been reported as effectors in fungi and oomycetes [53,54].

A putative serine protease (GenBank accession number AM411657) was identified among the *P. brassicae* genes that were expressed during infection [55]. This protease carried a predicted signal peptide sequence but lacked homologs in other plant pathogens. Further studies identified the serine protease as Pro1 [56], a member of the S28 family of proteases that, due to its proteolytic activity, may play a role during clubroot pathogenesis by stimulating resting spore germination [56]. Curiously, none of these studies suggested that this protein was an effector, probably because its suggested role occurs outside of the plant cell.

### Nuclear localization domains

Differing from many fungi and oomycetes, *P. brassicae* is an intracellular pathogen [57]. Another criterion used to identify putative effectors from obligate intracellular pathogens has been the presence of nuclear localization domains, which allows effectors to directly modulate plant gene expression [58,59]. For many years, effectors capable of migrating to the plant cell nucleus have only been described in bacteria [58–60], but more recently these motifs together with nuclear localization of effectors has been described in fungi [61,62] and nematodes [63]. In bioinformatics pipelines designed to identify putative effectors, the inclusion of steps to remove proteins containing subcellular localization signals will remove these effectors, although researchers could analyze these amino acid sequences directly using the online tool TargetP 1.1 Server (http://www.cbs.dtu.dk/services/TargetP). To date, none of the studies on the secretome and putative effectors of *P. brassicae* have detected or identified secreted proteins with such domains and none of the putative secreted proteins reported for *P. brassicae* have been identified as containing nuclear localization domains [20,21].

### Pexel motif

*P. brassicae* is evolutionarily closer to the malaria parasite, *Plasmodium spp*., than to oomycetes or fungal pathogens [5]. While there are many differences between the immune system of animals and plants, they both share the common characteristic of being targeted by pathogen effectors [64]. The discovery of the Pexel motif (Plasmodium export element) was a ground breaking finding that contributed to the understanding of the infection process of *Plasmodium* [65]. Pexel is a pentameric motif present in the N-terminal portion of all the proteins translocated through the parasitophorous vacuole membrane. It is comprised of a positively charged, hydrophilic amino acid in position one (Arg or Lys), a hydrophobic amino acid in position three (Leu or Ile), and another less conserved amino acid in position 5 (predominantly Asp, Glu, or Gln), with non-charged amino acids in positions two and four (Ser, Thr, Cys, Met, Asn, or Gln) [65]. The N-terminal domain of an effector protein from the soybean cyst nematode *Heterodera glycines*, containing unique sequence similarity to domains of an effector of *Plasmodium spp* [66]., indicates that the use of analogous effectors by highly diverse parasites of plants and animals occurs and is worth exploring.

### Plant pathogenic plasmodiophorids

*P. brassicae* is not the only plant pathogenic protist [67], nor is it the only plasmodiophorid affecting economically important crops: the group includes *Spongospora subterranean*, the causal agent of powdery scab on potato tubers, and *Polymyxa spp., Polymyxa graminis* and *Polymyxa betae*, which affect graminaceous plants and Chenopodiaceae plants, respectively [68,69].

Genomic sequences, although limited, are available from *S. subterranea* [70–72] and comprehensive *S. subterranea* transcriptomic datasets are available from root galls [5,20]. These data suggest intron-rich genes and an enrichment of chitin-related enzymes in the *S. subterranean* transcriptome. Transposable elements are more expressed in *S. subterranea* than in *P. brassicae* [20,70,71], but evidence for recombination in *S. subterranea* is limited and there is little understanding of sexual recombination in phytomyxids [73]. A study of secreted proteins in *S. subterranea* has been carried out, identifying the benzoic acid/salicylic acid methyltransferase-encoding gene over-expression that was previously described [20]. To date, there is no genomic data available for *Polymyxa spp* [67]..

## Life cycle and effectors

*P. brassicae* has three main stages to its life cycle, (*i*) survival in the soil and germination of resting spores, (*ii*) root hair or epidermal cell infection, and (*iii*) cortical infection (Figure 1B), although the pathogen has also been observed in phloem (Reviewed in Kageyama and Asano [7]). The serine protease, Pro1, is thought to be involved in step 1 (Figure 1B), the germination of resting spores [56]. From step 2 to 5 of the life cycle (Figure 1B), it is expected that other proteases and protease inhibitors, such as cys-rich proteases and methyltransferases will be produced to suppress plant immunity, thereby preventing the host plant from mounting responses such as programmed cell death and increasing the probability of success of the infection process [33]. During the formation of primary and secondary plasmodia, infected root tissues develop into swollen galls; it is expected that *P. brassicae* will secret an array of effector proteins triggering growth, expansion and differentiations of infected host cells. During zoospore maturation (Figure 1B), as expected, *P. brassica*e effectors with chitin-binding domains will express to remove chitin fragments that otherwise could trigger PAMP–associated immunity during late stages of the *P. brassicae* life cycle (Figure 1). Based on previous studies, might be also expected over expression of effectors implies on manipulating plant defense such as PbBSMT [36] and effectors disturbing plant meristematic activity during the formation of secondary zoospore and secondary plasmodia [74]. While there are still steps in the life cycle of *P. brassicae* that remain to be clarified, the study of effectors and their roles in life cycle completion and clubroot disease progression is an arena waiting to be explored.

## A coherent pipeline to identify effectors

**Figure 2.**
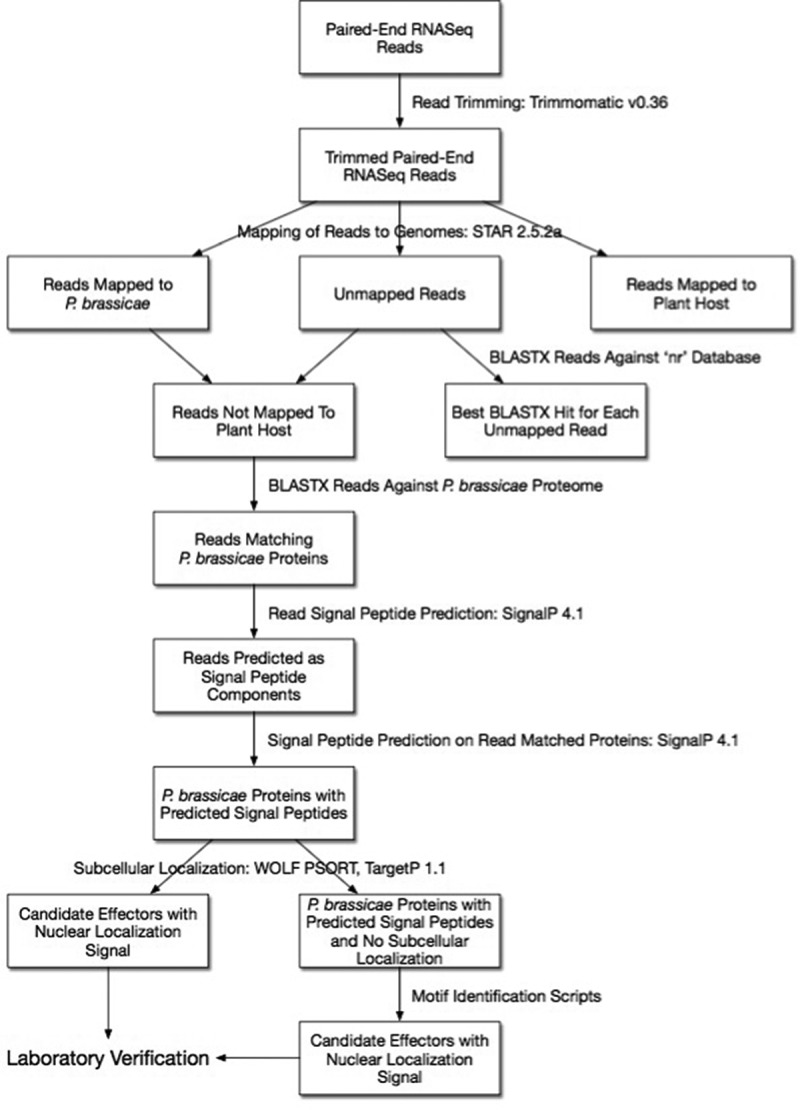
Coherent pipeline to identify putative effector proteins of *Plasmodiophora brassicae*. The pipeline assumes: (1) Researchers are starting with RNA-Seq reads from a host plant infected with *P. brassicae*; (2) The draft genomes available for *P. brassicae*, other plasmodiophorids, oomycetes, and other plant pathogens are used; (3) Motifs mentioned in the review or structural similarities with previously described effector proteins were identified.

Computational prediction is an excellent starting point to screen for putative effectors, to identify functional domains and to help us understand the evolution, distribution and characteristics of effectors [75,76]. Utilizing the information presented in this review we have designed a coherent pipeline aimed at identifying putative effectors involved in the infection process by *P. brassicae* and subsequent clubroot disease progression in its plant host (Figure 2). The pipeline makes use of tools to identify the origin of reads, and identify motifs and functions of the predicted secreted proteins. The bioinformatics tools referred to in the pipeline, together with their descriptions and websites, are listed in Table 2. This pipeline is only a suggestion based on our previous experience [77], and although key parameters should be set in order to run it properly, it is a good starting point.

**Table 2.** Bioinformatics tools used in a coherent pipeline to identify putative effectors of *P. brassicae.*

Bioinformatics tool	Description	Website	Reference
Trimmomatic	A fast, multithreaded command line tool that can be used to trim and crop Illumina data as well as to remove adapters.	http://www.usadellab.org/trimmomatic	^[80]^
STAR	An algorithm for aligning high-throughput long and short RNA-seq data to a reference genome.	http://code.google.com/p/rna-star	^[81]^
SignalP	Software that predicts signal peptide cleavage sites in proteins from eukaryotes and prokaryotes.	http://www.cbs.dtu.dk/services/SignalP	^[82]^
WoLF PSORT	An algorithm that predicts the subcellular localization sites of proteins based on their amino acid sequences. It makes predictions based on both known sorting signal motifs and some correlative sequence features such as amino acid content.	www.wolfpsort.org	^[83]^
TargetP	Tool that predicts subcellular location of eukaryotic proteins.	http://www.cbs.dtu.dk/services/TargetP	^[84]^

## Concluding remarks

Bioinformatics analysis is the route to the identification of candidate effector proteins, but laboratory confirmation of function will always be required. Validation of the RNA-seq data through qPCR [77] or ddPCR [78], identification of the subcellular localization of the candidate effector proteins [20] through transient expression or stable transformation [21,79] and identification of the plant proteins interacting with the pathogen effectors are some of the logical next steps towards the identification of *P. brassicae* effector proteins. The work with pathogen effectors in general is challenging, but the work with intracellular, biotrophic pathogens appears to be even more so, requiring creativity and novel solutions.

## References

[1] Hwang SF, Strelkov SE, Feng J, et al Plasmodiophora brassicae: a review of an emerging pathogen of the Canadian canola (Brassica napus) crop. Mol Plant Pathol. 2012;13:105–113. 21726396 10.1111/j.1364-3703.2011.00729.x PMC6638701

[2] Strelkov SE, Hwang SF. Clubroot in the Canadian canola crop: 10 years into the outbreak. Can J Plant Pathol. 2014;36(S1):27–36.

[3] Bhering AS, Do Carmo MGF, Matos TM, et al Soil factors related to the severity of clubroot in Rio de Janeiro. Brazil Plant Disease. 2017;101:1345–1353. 10.1094/PDIS-07-16-1024-SR 30678583

[4] Aist JR, Williams PH The cytology and kinetics of cabbage root hair penetration by *Plasmodiophora brassicae*. Can J Bot. 1971;49:2023–2034.

[5] Burki F, Kudryavtsev A, Matz MV, et al Evolution of Rhizaria: new insights from phylogenomic analysis of uncultivated protists. BMC Evol Biol. 2010;10:377. 21126361 10.1186/1471-2148-10-377 PMC3014934

[6] Burki F, Keeling PJ Rhizaria. Curr Biol. 2014;24(3):R103–7. 24502779 10.1016/j.cub.2013.12.025

[7] Kageyama K, Asano T Life cycle of Plasmodiophora brassicae. J Plant Growth Regul. 2009;28(3):203–211.

[8] Peng G, Lahlali R, Hwang SF, et al Crop rotation, cultivar resistance, and fungicides/biofungicides for managing clubroot (Plasmodiophora brassicae) on canola. Can J Plant Pathol. 2014;36(S1):99–112.

[9] Rahman H, Bennett RA, Seguin-Swartz G Broadening genetic diversity in Brassica napus canola: development of canola-quality spring B. napus from B. napus × B. oleracea var. alboglabra interspecific crosses. Can J Plant Sci. 2014;95(1):29–41.

[10] Fredua-Agyeman R, Hwang S-F, Strelkov SE, et al Assessment of resistance to ‘new’ virulent populations of Plasmodiophora brassicae reveals potential loss of clubroot resistance genes from donor parent *Brassica rapa* L. ssp. rapifera (ECD 04) during doubled haploid production. Mol Plant Pathol. 2017 DOI:10.1111/ppa.12816

[11] Rahman H, Shakir A, Hassan MJ Breeding for clubroot resistant spring canola (Brassica napus L.) for the Canadian prairies: can the European winter canola cv. ‘Mendel’ be used as a source of resistance? Can J Plant Sci. 2011;91:447–458.

[12] Matsumoto E, Ueno H, Aruga D, et al Accumulation of three clubroot resistance genes through marker-assisted selection in Chinese Cabbage (Brassica rapa ssp. pekinensis). J Jpn Soc Hortic Sci. 2012;81:184–190.

[13] Diederichsen E, Frauen M, Ludwig-Müller J Clubroot disease management challenges from a German perspective. Can J Plant Pathol. 2014;36:85–98.

[14] Ueno H, Matsumoto E, Aruga D, et al Molecular characterization of the CRa gene conferring clubroot resistance in Brassica rapa. Plant Mol Biol. 2012;80:621–629. 23054353 10.1007/s11103-012-9971-5

[15] Hatakeyama H, Suwabe K, Tomita RN, et al Identification and characterization of Crr1a, a gene for resistance to clubroot disease (Plasmodiophora brassicae Woronin) In Brassica rapa L. PLoS ONE. 2013;8:e54745. 23382954 10.1371/journal.pone.0054745 PMC3559844

[16] Figdore SS, Ferreira ME, Slocum MK, et al Association of RFLP markers with trait loci affecting clubroot resistance and morphological characters in Brassica oleracea L. Euphytica. 1993;69:33–44.

[17] Rocherieux J, Glory P, Giboulot A, et al Isolate-specific and broad-spectrum QTLs are involved in the control of clubroot in Brassica oleracea. Theor Appl Genet. 2004;108:1555–1563. 15007504 10.1007/s00122-003-1580-x

[18] Nagaoka T, Doullah MA, Matsumoto S, et al Identification of QTLs that control clubroot resistance in Brassica oleracea and comparative analysis of clubroot resistance genes between *B. rapa* and *B. oleracea*. Theor Appl Genet. 2010;120:1335–1346. 20069415 10.1007/s00122-010-1259-z

[19] Lee J, Izzah NK, Choi BS, et al Genotyping-by-sequencing map permits identification of clubroot resistance QTLs and revision of the reference genome assembly in cabbage (Brassica oleracea L.). DNA Res. 2015;23:29–41. 26622061 10.1093/dnares/dsv034 PMC4755525

[20] Schwelm A, Fogelqvist J, Knaust A, et al The Plasmodiophora brassicae genome reveals insights in its life cycle and ancestry of chitin synthases. Sci Rep. 2015;5:11153. 26084520 10.1038/srep11153 PMC4471660

[21] Rolfe SA, Strelkov SE, Links MG, et al. The compact genome of the plant pathogen Plasmodiophora brassicae is adapted to intracellular interactions with host Brassica spp. BMC Genomics. 2016;17:272. 27036196 10.1186/s12864-016-2597-2 PMC4815078

[22] Vleeshouwers VGAA, Oliver RP Effectors as tools in disease resistance breeding against biotrophic, hemibiotrophic, and necrotrophic plant pathogens. Molecular Plant-Microbe Interactions. 2014;27(3):196–206. 24405032 10.1094/MPMI-10-13-0313-IA

[23] Cano LM, Raffaele S, Haugen RH, et al Major transcriptome reprogramming underlies floral mimicry induced by the rust fungus Puccinia monoica in Boechera *stricta*. PLoS One. 2013;8:e75293. 24069397 10.1371/journal.pone.0075293 PMC3775748

[24] Sugio A, Kingdom HN, MacLean AM, et al Phytoplasma protein effector SAP11 enhances insect vector reproduction by manipulating plant development and defense hormone biosynthesis. PNAS. 2011;108(48):E1254–E1263. 22065743 10.1073/pnas.1105664108 PMC3228479

[25] Nemri A, Saunders DGO, Anderson C, et al The genome sequence and effector complement of the flax rust pathogen Melampsora lini. Front Plant Sci. 2014;5 DOI:10.3389/fpls.2014.00098. PMC3970004 24715894

[26] Dodds PN, Lawrence GJ, Catanzariti A-M, et al The Melampsora lini AvrL567 avirulence genes are expressed in haustoria and their products are Recognized inside plant cells. Plant Cell. 2004;16:755–768. 14973158 10.1105/tpc.020040 PMC385286

[27] Van Esse HP, Bolton MD, Stergiopoulos I, et al The chitin-binding Cladosporium fulvum effector protein Avr4 is a virulence factor. Molecular Plant-Microbe Interactions. 2007;20(9):1092–1101. 17849712 10.1094/MPMI-20-9-1092

[28] Salcedo A, Rutter W, Wang S, et al Variation in the AvrSr35 gene determines *Sr35* resistance against wheat stem rust race Ug99. Science. 2017;358:1604–1606. 29269474 10.1126/science.aao7294 PMC6518949

[29] Chen J, Upadhyaya NM, Ortiz D, et al Loss of AvrSr50 by somatic exchange in stem rust leads to virulence for Sr50 resistance in wheat. Science. 2017;358:1607–1610. 29269475 10.1126/science.aao4810

[30] Mago R, Zhang P, Vautrin S, et al The wheat Sr50 gene reveals rich diversity at a cereal disease resistance locus. Nat Plants. 2015;1:15186. 27251721 10.1038/nplants.2015.186

[31] Dodds PN, Rathjen JP Plant immunity: towards an integrated view of plant– pathogen interactions. Nat Rev Genet. 2010;11(8):539–548. 20585331 10.1038/nrg2812

[32] Jones JDG, Dangl JL The plant immune system. Nature. 2006;444:323–329. 17108957 10.1038/nature05286

[33] Siemens J, González MC, Wolf S, et al Extracellular invertase is involved in the regulation of clubroot disease in Arabidopsis thaliana. Mol Plant Pathol. 2011;12:247–262. 21355997 10.1111/j.1364-3703.2010.00667.x PMC6640435

[34] Duplessis S, Cuomo CA, Lin Y-C, et al Obligate biotrophy features unravelled by the genomic analysis of rust fungi. Proc Natl Acad Sci USA. 2011;108:9166–9171. 21536894 10.1073/pnas.1019315108 PMC3107277

[35] Hacquard S, Joly DL, Lin Y-C, et al A comprehensive analysis of genes encoding small secreted proteins identifies candidate effectors in Melampsora larici-populina (poplar leaf rust). Molecular Plant Microbe Interactions. 2012;25:279–293. 22046958 10.1094/MPMI-09-11-0238

[36] Ludwig-Müller J, Jülken S, Geib K, et al A novel methyltransferase from the intracellular pathogen Plasmodiophora brassicae methylates salicylic acid. Mol Plant Pathol. 2015;16(4):349–364. 25135243 10.1111/mpp.12185 PMC6638400

[37] Dempsey DA, Vlot AC, Wildermuth MC, et al Salicylic acid biosynthesis and metabolism In: Torii K, editor. The Arabidopsis Book. Vol. 9 Rockville, MD: The American Society of Plant Biologists; 2011 p. e0156. 10.1199/tab.0156 PMC3268552 22303280

[38] Seskar M, Shulaev V, Raskin I Endogenous methyl salicylate in pathogen-inoculated tobacco plants. Plant Physiol. 1998;116:387–392.

[39] Djamei A, Schipper K, Rabe F, et al Metabolic priming by a secreted fungal effector. Nature. 2011;478:395–398. 21976020 10.1038/nature10454

[40] Lo Presti L, Lanver D, Schweizer G, et al Fungal effectors and plant susceptibility. Annu Rev Plant Biol. 2015;66:513–545. 25923844 10.1146/annurev-arplant-043014-114623

[41] Stergiopoulos I, De Wit PJGM Fungal effector proteins. Annu Rev Phytopathol. 2009;47:233–263. 19400631 10.1146/annurev.phyto.112408.132637

[42] De Carvalho M, Nascimento LC, Darben LM, et al Prediction of the in planta Phakopsora pachyrhizi secretome and potential effector families. Mol Plant Pathol. 2017;18(3):363–377. 27010366 10.1111/mpp.12405 PMC6638266

[43] Rafiqi M, Gan PHP, Ravensdale M, et al Internalization of flax rust avirulence proteins into flax and tobacco cells can occur in the absence of the pathogen. Plant Cell. 2010;22:2017–2032. 20525849 10.1105/tpc.109.072983 PMC2910983

[44] Yaeno T, Li H, Chaparro-Garcia A, et al Phosphatidyl inositol monophosphate-binding interface in the oomycete RXLR effector AVR3a is required for its stability in host cells to modulate plant immunity. Proc Natl Acad Sci USA. 2011;108:14682–14687. 21821794 10.1073/pnas.1106002108 PMC3167543

[45] Kale SD, Gu B, Capelluto DG, et al External lipid PI3P mediates entry of eukaryotic pathogen effectors into plant and animal host cells. Cell. 2010;142:284–295. 20655469 10.1016/j.cell.2010.06.008

[46] Xiang J, Li X, Yin L, et al A candidate RxLR effector from Plasmopara viticola can elicit immune responses in Nicotiana benthamiana. BMC Plant Biology. 2017;17:75. 28410577 10.1186/s12870-017-1016-4 PMC5391559

[47] Wirthmueller L, Maqbool A, Banfield MJ On the front line: structural insights into plant-pathogen interactions. Nat Rev Microbiol. 2013;11:761–776. 24100360 10.1038/nrmicro3118

[48] Van Den Burg HA, Spronk CAEM, Boeren S, et al Binding of the AVR4 elicitor of Cladosporium fulvum to chitotriose units is facilitated by positive allosteric protein- protein interactions: the chitin-binding site of Avr4 represents a novel binding site on the folding scaffold shared between the invertebrate and the plant chitin-binding domain. J Biol Chem. 2004;279:16786–16796. 14769793 10.1074/jbc.M312594200

[49] Van Den Burg HA, Harrison SJ, Joosten MHAJ, et al Cladosporium fulvum Avr4 protects fungal cell walls against hydrolysis by plant chitinases accumulating during infection. Molecular Plant-Microbe Interaction. 2006;19:1420–1430. 10.1094/MPMI-19-1420 17153926

[50] Bolton MD, Van Esse HP, Vossen JH, et al The novel Cladosporium fulvum lysin motif effector Ecp6 is a virulence factor with orthologues in other fungal species. Mol Microbiol. 2008;69:119–136. 18452583 10.1111/j.1365-2958.2008.06270.x

[51] Spanu PD, Abbott JC, Amselem J, et al Genome expansion and gene loss in powdery mildew fungi reveal trade offs in extreme parasitism. Science. 2010;330:1543–1546. 21148392 10.1126/science.1194573

[52] Rooney HCE, Van’t Klooster JW, Van Der Hoorn RAL, et al Cladosporium Avr2 inhibits tomato Rcr3 protease required for Cf-2-dependent disease resistance. Science. 2005;308:1783–1786. 15845874 10.1126/science.1111404

[53] Pallaghy PK, Nielsen KJ, Craik DJ, et al A common structural motif incorporating a cysteine knot and a triple-stranded Î^2^-sheet in toxic and inhibitory polypeptides. Protein Sci. 1994;3:1833–1839. 7849598 10.1002/pro.5560031022 PMC2142598

[54] Van Den Hooven HW, Van Den Burg HA, Vossen P, et al Disulfide bond structure of the AVR9 elicitor of the fungal tomato pathogen Cladosporium fulvum: evidence for a cystine knot. Biochemistry. 2001;40:3458–3466. 11297411 10.1021/bi0023089

[55] Bulman S, Siemens J, Ridgway HJ, et al Identification of genes from the obligate intracellular plant pathogen, Plasmodiophora brassicae. FEMS Microbiol Lett. 2006;264:198–204. 17064373 10.1111/j.1574-6968.2006.00466.x

[56] Feng J, Hwang RU, Hwang S-F, et al Molecular characterization of a serine protease Pro1 from Plasmodiophora brassicae that stimulates resting spore germination. Plant Pathology. 2010;11(4):503–512. 10.1111/j.1364-3703.2010.00623.x PMC6640502 20618708

[57] Moxham SE, Buczacki ST Structure of the resting spore wall of Plasmodiophora brassicae revealed by electron microscopy and chemical digestion. Trans Br Mycological Soc. 1983;81:221–231.

[58] Gurlebeck D, Thieme F, Bonas U Type III effector proteins from the plant pathogen Xanthomonas and their role in the interaction with the host plant. J Plant Physiol. 2006;163:233–255. 16386329 10.1016/j.jplph.2005.11.011

[59] Poueymiro M, Genin S Secreted proteins from Ralstonia solanacearum: a hundred tricks to kill a plant. Curr Opin Microbiol. 2009;12:44–52. 19144559 10.1016/j.mib.2008.11.008

[60] Bai X, Correa VR, Toruño TY, et al AY-WB phytoplasma secretes a protein that targets plant cell nuclei. Molecular Plant Microbe Interaction. 2009;22:18–30. 10.1094/MPMI-22-1-0018 19061399

[61] Caillaud M-C, Piquerez SJM, Fabro G, et al Subcellular localization of the Hpa RxLR effector repertoire identifies a tonoplast-associated protein HaRxL17 that confers enhanced plant susceptibility. Plant J. 2012;69:252–265. 21914011 10.1111/j.1365-313X.2011.04787.x

[62] Vargas WA, Sanz-Martín JM, Rech GE, et al A fungal effector with host nuclear localization and DNA-binding properties is required for maize anthracnose development. Molecular Plant Microbe Interaction. 2016;29:83–95. 10.1094/MPMI-09-15-0209-R 26554735

[63] Quentin M, Abad P, Favery B Plant parasitic nematode effectors target host defense and nuclear functions to establish feeding cells. Front Plant Sci. 2013;4:53. 23493679 10.3389/fpls.2013.00053 PMC3595553

[64] Espinosa A, Alfano JR Disabling surveillance: bacterial type III secretion system effectors that suppress innate immunity. Cell Microbiol. 2004;6:1027–1040. 15469432 10.1111/j.1462-5822.2004.00452.x

[65] Marti M, Good RT, Rug M, et al Targeting malaria virulence and remodeling proteins to the host erythrocyte. Science. 2004;306:1930–1933. 15591202 10.1126/science.1102452

[66] Noon JB, Qi M, Sill1 DN, et al A Plasmodium-like virulence effector of the soybean cyst nematode suppresses plant innate immunity. New Phytologist. 2016;212:444–460. 27265684 10.1111/nph.14047

[67] Schwelm A, Badstöber J, Bulman S, et al Not in your usual Top 10: protists that infect plants and algae. Mol Plant Pathol. 2017 DOI:10.1111/mpp.12580 PMC5772912 29024322

[68] Legreve A, Vanpee B, Delfosse P, et al Host range of tropical and sub-tropical isolates of *Polymyxa graminis*. Eur J Plant Pathol. 2000;106:379–389.

[69] Legreve A, Delfosse P, Maraite H Phylogenetic analysis of Polymyxa species based on nuclear 5.8S and internal transcribed spacers ribosomal DNA sequences. Mycology Res. 2002;106:138–147.

[70] Bulman S, Candy JM, Fiers M, et al Genomics of biotrophic, plant-infecting plasmodiophorids using in vitro dual cultures. Protist. 2011;162:449–461. 21183405 10.1016/j.protis.2010.09.004

[71] Gutiérrez PA, Alzate JF, Montoya MM Analysis of carbohydrate metabolism genes of Spongospora subterranea using 454 pyrosequencing. Revista Facultad Nacional De Agronomía Medellín. 2014;67:7247–7260.

[72] Gutiérrez P, Bulman S, Alzate JF, et al Mitochondrial genome sequence of the potato powdery scab pathogen *Spongospora subterranea*. Mitochondrial DNA. Part A, DNA Mapping, Sequencing, and Analysis. 2016;27:58–59. 10.3109/19401736.2013.873898 24438302

[73] Bulman S, Braselton JP Rhizaria: phytomyxea In: (Mclaughlin DJ, Spatafora JW, editors. The Mycota VII, Part A, Systematics and Evolution. Berlin (Germany): Springer-Verlag; 2014 p. 99–112.

[74] Malinowski R, Smith JA, Fleming AJ, et al Gall formation in clubroot-infected Arabidopsis results from an increase in existing meristematic activities of the host but is not essential for the completion of the pathogen life cycle. Plant J. 2012;71:226–238. 22394393 10.1111/j.1365-313X.2012.04983.x

[75] Zuccaro A, Lahrmann U, GüLdener U, et al Endophytic life strategies decoded by genome and transcriptome analyses of the mutualistic root symbiont Piriformospora indica. PLoS Pathog. 2011;7:e1002290. 22022265 10.1371/journal.ppat.1002290 PMC3192844

[76] Ye W, Wang Y, Wang Y Bioinformatics analysis reveals abundant short alpha-helices as a common structural feature of oomycete RxLR effector proteins. PLoSONE. 2015;10:e0135240. 10.1371/journal.pone.0135240 PMC4529148 26252511

[77] Irani S, Trost B, Waldner M, et al Transcriptome analysis of response to Plasmodiophora brassicae infection in the Arabidopsis shoot and root. BMC Genomics. 2018;19:23. 29304736 10.1186/s12864-017-4426-7 PMC5756429

[78] Seifbarghi S, Borhan MH, Wei Y, et al Changes in the Sclerotinia sclerotiorum transcriptome during infection of Brassica napus. BMC Genomics. 2017;18:266. 28356071 10.1186/s12864-017-3642-5 PMC5372324

[79] Bulman S, Richter F, Marschollek S, et al Arabidopsis thaliana expressing PbBSMT, a gene encoding a SABATH-type methyltransferase from the plant pathogenic protist Plasmodiophora brassicae, show leaf chlorosis and altered host susceptibility. Plant Biol. 2018 DOI:10.1111/plb.12728 29607585

[80] Bolger AM, Lohse M, Usadel B Trimmomatic:A flexible trimmer for Illumina Sequence Data. Bioinformatics. 2014;30(15):2114–2120.. 10.1093/bioinformatics/btu170 PMC4103590 24695404

[81] Dobin A, Davis CA, Schlesinger F, et al STAR: ultrafast universal RNA-seq aligner. Bioinformatics. 2013;29:15–21. 23104886 10.1093/bioinformatics/bts635 PMC3530905

[82] Petersen TN, Brunak S, Von Heijne G, et al SignalP4.0: discriminating signal peptides from transmembrane regions. Nat Methods. 2011;8:785–786. 21959131 10.1038/nmeth.1701

[83] Horton P, Park K-J, Obayashi T, et al WoLF PSORT: protein localization predictor. Nucleic Acids Res. 2007;35:W585–W587. 17517783 10.1093/nar/gkm259 PMC1933216

[84] Emanuelsson O, Nielsen H, Brunak S, et al Predicting subcellular localization of proteins based on their N-terminal amino acid sequence. J Mol Biol. 2000;300:1005–1016. 10891285 10.1006/jmbi.2000.3903

